# A first insight into tuberculosis transmission at the border of Ecuador and Colombia: a retrospective study of the population structure of *Mycobacterium tuberculosis* in Esmeraldas province

**DOI:** 10.3389/fpubh.2024.1343350

**Published:** 2024-02-07

**Authors:** Bernardo Castro-Rodriguez, Sebastián Espinoza-Andrade, Greta Franco-Sotomayor, José Manuel Benítez-Medina, Natalia Jiménez-Pizarro, Camilo Cárdenas-Franco, Juan Carlos Granda, Jose Luis Jouvin, Solon Alberto Orlando, Javier Hermoso de Mendoza, Miguel Ángel García-Bereguiain

**Affiliations:** ^1^One Health Research Group, Universidad de las Américas, Quito, Ecuador; ^2^Instituto Nacional de Investigación en Salud Pública “Leopoldo Izquieta Pérez”, Guayaquil, Ecuador; ^3^Facultad de Medicina, Universidad Católica Santiago de Guayaquil, Guayaquil, Ecuador; ^4^Departamento de Patología Infecciosa, Facultad de Veterinaria, Universidad de Extremadura, Cáceres, Spain; ^5^Universidad Espíritu Santo, Guayaquil, Ecuador

**Keywords:** *Mycobacterium tuberculosis*, MIRU-VNTR, spoligotyping, migration, Ecuador

## Abstract

**Objective:**

Tuberculosis (TB) is a major public health concern in Ecuador and Colombia, considering that both countries are high-burden TB settings. Molecular epidemiology is crucial to understand the transmission dynamics of *Mycobacterium tuberculosis* complex (MTBC) and to identify active transmission clusters of regional importance.

**Methods:**

We studied the potential transmission of TB between Colombia and Ecuador through the analysis of the population structure of MTBC lineages circulating in the Ecuadorian province of Esmeraldas at the border with Colombia. A total of 105 MTBC strains were characterized by 24-loci MIRU-VNTR and spoligotyping.

**Results:**

MTBC lineage 4 is only present in Esmeraldas; no MTBC strains belonging to Lineage 2–sublineage Beijing were found despite its presence in other provinces of Ecuador and, in Colombia. Genotyping results revealed a high degree of diversity for MTBC in Esmeraldas: Neither active transmission clusters within this province nor including MTBC strains from Colombia or other provinces of Ecuador were found.

**Conclusion:**

Our data suggest that tuberculosis dynamics in this rural and isolated area may be not related to highly transmitted strains but could be influenced by other health determinants that favor TB relapse such as poverty and poor health system access. Further studies including a larger number of MTBC strains from Esmeraldas are necessary to test this hypothesis.

## Introduction

The *Mycobacterium tuberculosis* complex (MTBC) includes bacteria with specific microbiological characteristics that confer certain properties for host adaptation, virulence, and transmission, thus contributing to the development of tuberculosis (TB), an infectious disease that mainly spreads by close person-to-person contact through expulsion and inhalation of contaminated aerosols, affecting the lungs or other organs (1, 2). Considering the importance of TB as one of the leading causes of death around the world, behind COVID-19 and above HIV-AIDS, the understanding of the genetic diversity of *M. tuberculosis* (MTB) is critical for TB surveillance and prevention (1, 3). Briefly, the MTBC is comprised of seven highly related lineages that differ in geographical distribution, infectious capabilities, transmission modes, and resistance to antibiotics: Lineage 1 (Indo-Oceanic), Lineage 2 (East Asia), Lineage 3 (India–East Asia), Lineage 4 (Euro-American), Lineage 5 (West African 1), Lineage 6 (West African 2), and Lineage 7 (Ethiopia). The most significant sublineages are lineage 1, MANU and EAI; lineage 2, Beijing; lineage 3, Central Asian (CAS) and Delhi; lineage 4, Haarlem, Latin American Mediterranean (LAM), T, X, S, Ghana, URAL, TUR, Uganda, and H37Rv; and lineage 6, AFRI and West African (3, 4). Therefore, the use of molecular markers present in the genome of *M. tuberculosis*, such as mycobacterial interspersed repetitive units (MIRU-VNTR typing method) and/or spacer sequences in the direct repeat (DR) region (spoligotyping method), allows the characterization of transmission dynamics and clusters of MTBC strains (5–7).

For 2021, the World Health Organization (WHO) estimated for Ecuador a burden of 8500 TB cases (rate of 48/100,000 population) and 370 cases of multidrug resistance TB (MDR-TB) (rate 2.1 of 100,000 population), 830 deaths of HIV-negative TB patients and 330 deaths of HIV-positive TB patients (TB case fatality ratio: 14%) (8). MTBC molecular epidemiology studies in Ecuador are really scarce (9–12). There is a single study addressing the population structure and genetic diversity of MTBC in the whole country, showing that MTBC lineage 4 sublineage LAM is predominant countrywide, and sublineages X and S are also predominant in the Coastal and Andean regions, respectively (9). Similar results have been reported in other studies with MTBC strains from Quito (10) and Guayaquil (12).

For the same year 2021, the estimates of TB cases for Colombia provided by WHO were 21,000 (rate 41 of 100,000 population) and 1,100 cases of MDR-TB (rate 2.2 of 100,000 population), 1700 deaths of HIV-negative TB individuals, and 840 deaths of HIV-positive TB individuals (TB case fatality ratio: 12%) (13). However, total notified TB cases in both countries are lower than the estimates provided by the WHO, with 5,595 cases notified in Ecuador and 13,659 cases in Colombia, suggesting an underestimation of TB cases in both countries (8, 13, 14). The most predominant MTBC lineage in Colombia is lineage 4 (including sublineages like LAM, Haarlem, X, and S) but also lineage 2–sublineage Beijing (4, 15). Colombia and Peru have a significantly higher presence of MTBC lineage 2–sublineage Beijing than other countries in South America, representing a potential risk for TB control in the region (4, 15–22).

As neighbor countries and members of the Andean Community, Ecuador and Colombia have a historically intense migration flow (9). Considering that both countries are high-burden TB settings, and also that Colombia is a hot spot for MTBC lineage 2-Beijing (11, 23, 24), TB transnational transmission studies could improve strategies for TB control (9, 11). There is a previous study addressing the population structure of MTBC in Ecuador that reported clonal complexes formed by MTBC strains from Ecuador and Colombia, although no active transmission clusters were found (9). However, MTBC strains from the northern provinces of Ecuador on the border with Colombia were underrepresented in this study, so the existence of active transmission clusters could not be totally ruled out (9).

The goal of this retrospective study was to assess the population structure of MTBC in the province of Esmeraldas on the border of Ecuador and Colombia to analyze the TB transmission between those countries.

## Materials and methods

### *Mycobacterium tuberculosis* strains included in the study

A collection of 105 MTBC isolates from years 2014 to 2016 stored at “Instituto Nacional de Salud Pública e Investigación Leopoldo Izquieta Pérez” (INSPI) in Guayaquil (Ecuador) was included in the study, distributed in 42, 16, and 47 samples for 2014, 2015, and 2016, respectively. MTBC isolates are routinely processed at INSPI laboratories, where culture and antibiotic resistance profiling for first- and second-line drugs used in TB therapy is performed for MTBC cultures following Pan American Health Organization guidelines (25, 26). The samples were previously inactivated and stored to follow the guidelines from this government center. MTBC culture manipulation prior to inactivation was carried out in a BSL2+ facility.

This collection of 105 MTBC isolates included all the MTBC strains available at the time this study was carried out for Esmeraldas, located in the Northern Coastal Region of Ecuador, that borders with Colombia. Nevertheless, according to the reports from the Ecuadorian Ministry of Health for TB cases distributions by province, there were 121 and 200 TB cases reported in Esmeraldas for the years 2017 and 2018, respectively.^1^ If we estimate an average of 200 TB cases per year in this province, the total number of cases for 2014–2016 would be 600, so a collection of 105 MTBC strains would represent 17.5% of the total cases in Esmeraldas province.

Additionally, information from MTBC strains from Ecuador and Colombia was retrieved from the bibliography, as it has been done in similar studies (27, 28). For the phylogenetic analysis described below, 190 MTBC strains from Colombia for the years 2012–2014 (4) and 385 MTBC strains from Ecuador from the years 2012–2016 (9, 26) were included in the study.

#### *Mycobacterium tuberculosis* heat inactivation and DNA isolation

A sample from cultures of MTBC was collected and resuspended in TE buffer (10 mM Tris–HCl, 1 mM EDTA, pH 8.0), then inactivated by boiling at 95°C for 45 min. After this process, samples were centrifuged for 5 min at 10000 g and the supernatant was directly used for genotyping (11, 30).

#### MIRU-VNTR genotyping of MTBC strains

The method is PCR-based and allows the detection of different mycobacterial interspersed repetitive units (MIRU) located at multiple loci in the MTBC genome. Each MIRU allele is identified by a number, thus generating a numerical profile that is used for genotyping studies (5, 31, 32). Amplicons were observed in 2% UltraPure™ Agarose (Invitrogen, California, USA) gels of 15 cm x 10 cm in 0.5X Tris-boric acid-EDTA (TBE) buffer at 100 V for 3 h using a ladder 100-bp Plus Opti-DNA Marker (Cat.No.: G016, Applied Biological Materials Inc., British Columbia, Canada) for size determination. MIRU allele identification was performed according to Supply et al. (33).

#### *Mycobacterium tuberculosis* complex strains spoligotyping

Spoligotyping was performed as described elsewhere (34, 35). The results were compared to the databases available in the following free sites: the SITVIT2 website (http://www.pasteur-guadeloupe.fr:8081/SITVIT2/) and the MIRU-VNTRplus (https://www.miru-vntrplus.org/MIRU/index.faces).

#### *Phylogenetic analysis* of MTB strains

For phylogenetic analysis, 24 MIRU-VTNR and spoligotyping patterns belonging to the 105 MTBC strains from the years 2014–2016 from Esmeraldas were used. In addition, we retrieved information on MTBC strains from Ecuador and Colombia from the literature: (1) 24 MIRU-VTNR patterns of 385 MTBC isolates for years 2012–2016 from Ecuador (9, 29); (2) 24 MIRU-VTNR and spoligotyping patterns of 190 MTBC strains for years 2012–2014 from Colombia (4).

Genotyping data were analyzed using the MIRU-VNTRplus web application^2^ (36). Lineage identification was performed by similarity search, using MIRU-VNTR and spoligotyping information, by categorical distance measure (MIRU-VNTR weight: 1, Spoligo weight: 1). Calculation of Neighbor-joining Tree (NJT) and Minimum Spanning Tree (MST) were performed using MIRU-VNTR and/or Spoligotyping information when available.

## Results

### Drug susceptibility testing of MTBC strains from Esmeraldas Province of Ecuador

Regarding the 105 MTBC strains included in the study, the information for drug susceptibility testing was available for 98 of them: 23 of 98 (23.5%) were resistant to isoniazid (7 of 23 were monoresistant), 10 of 98 (10.2%) were resistant to streptomycin (6 of 10 were monoresistant), 6 of 98 (6.1%) were resistant to ethambutol, 17 of 98 (17.3%) were resistant to rifampicin (1 of 17 was monoresistant), and 5 of 28 (5.1%) were resistant to pyrazinamide. MTBC strains resistant to isoniazid and rifampicin (MDR) were 16 of 98 (16.3%). On the other hand, 71 of 98 (72.4%) MTBC strains were sensible to all the drugs tested (Table 1).

**Table 1:** Drug resistance profile for MTBC strains from Esmeraldas included in this study (MDR-TB are strains resistant to isoniazid + rifampicin).

Drug-resistant Profile	Isolates	Prevalence (%)
Isoniazid resistant	23	23.5
Streptomycin resistant	10	10.2
Ethambutol resistant	6	6.1
Rifampicin resistant	17	17.3
Pyrazinamide resistant	5	5.1
MDR-TB	16	16.3
Sensible to all tested drugs	71	72.4
Total	98	
Without information	7	6.7

### Population structure of MTBC strains from Esmeraldas province in Ecuador

**Figure 1:**
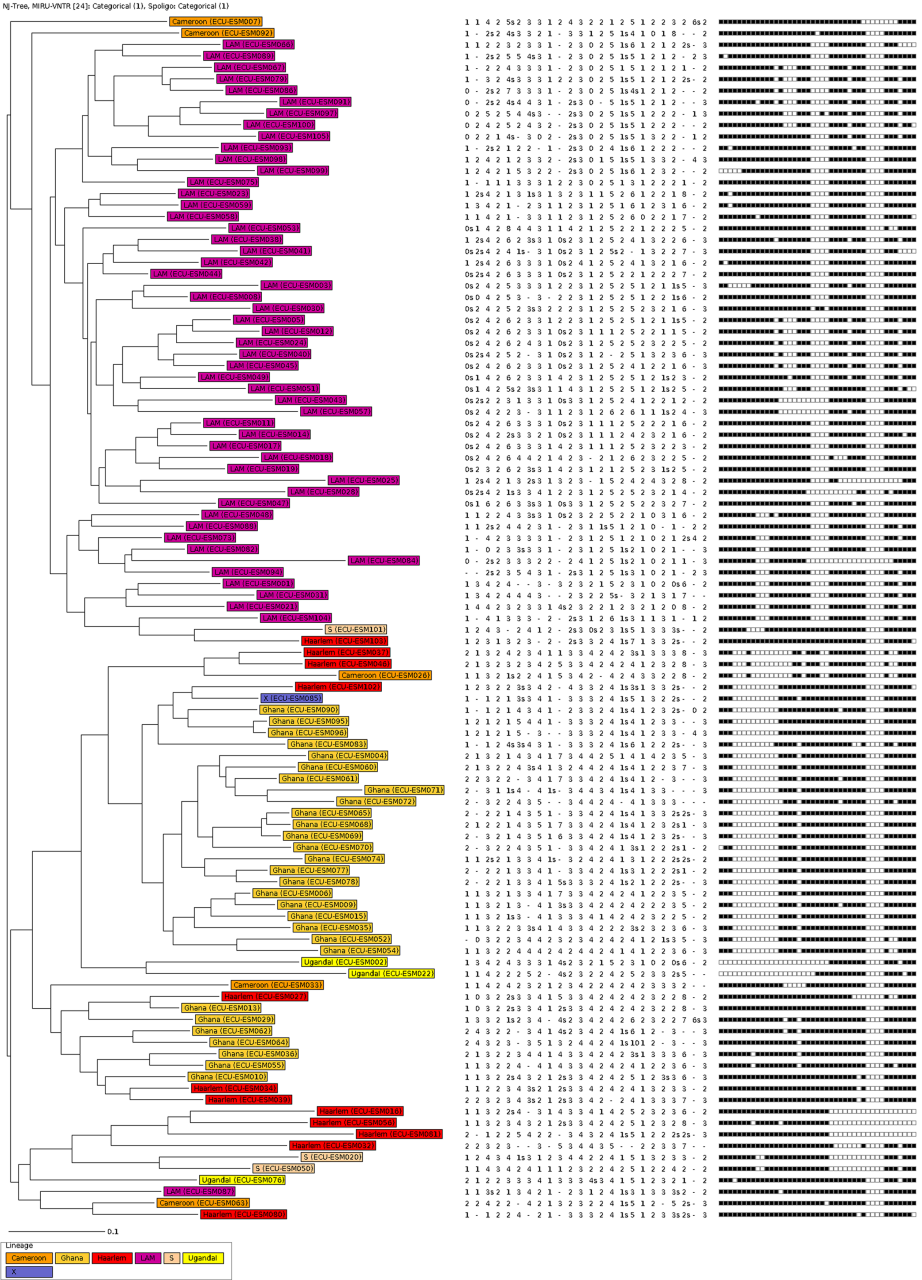
Population structure of MTBC from Esmeraldas province. The neighbor-joining tree was done with 24-loci MIRU-VNTR and spoligotyping data.

**Figure 2:**
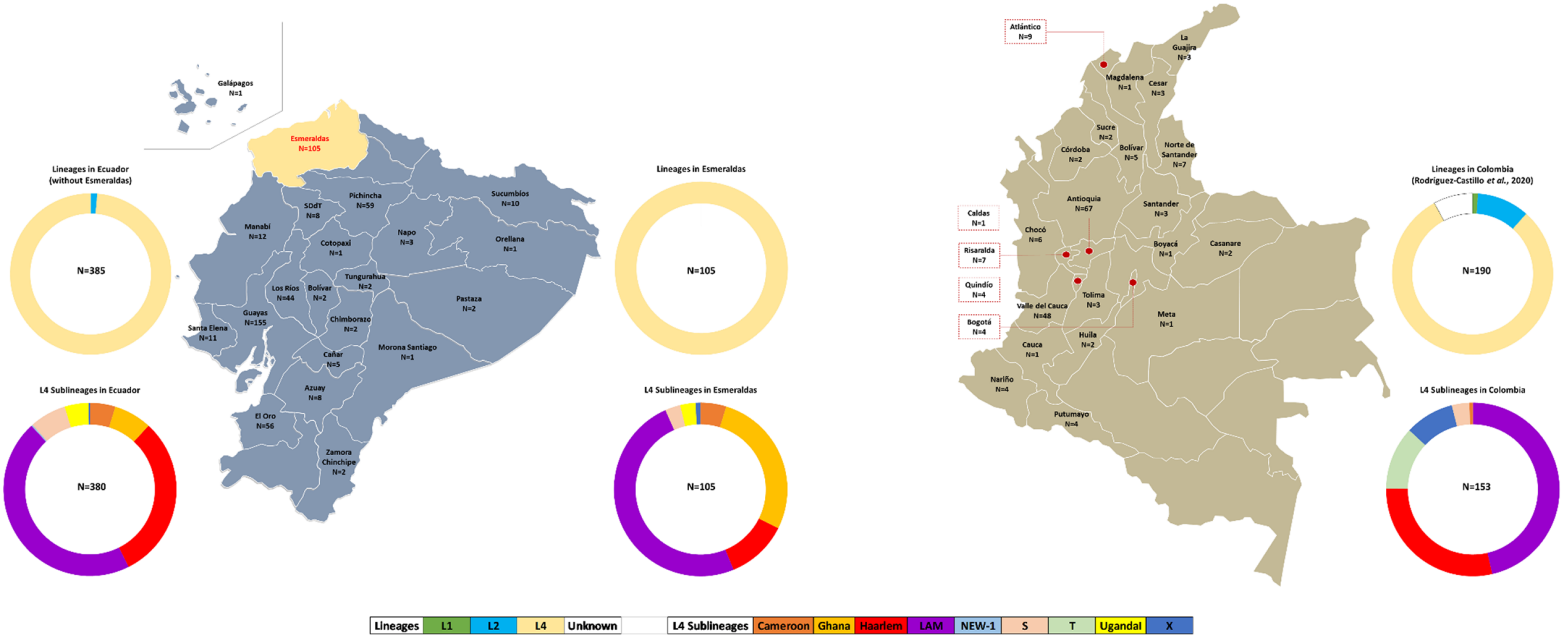
Geographical and sublineage distribution of MTBC strains from Esmeraldas (our study), Ecuador (9), and Colombia (20). Ring charts represent the occurrence of MTBC lineages in Esmeraldas province, the rest of Ecuador, and Colombia.

The 105 MTBC strains from Esmeraldas were analyzed using 24-loci MIRU-VNTR and spoligotyping. A neighbor-joining tree (NJT) based on this genotypic information is shown in Figure 1. The lineage distribution obtained for MTBC strains from Esmeraldas revealed a high degree of diversity, and no active transmission clusters without any single loci variation in 24-loci MIRU-VNTR were found (Figure 1). The lineage distribution for the MTBC strains from Esmeraldas province was 100% L4, with sublineages LAM 52/105 (49.5%), Ghana 29/105 (27.6%), Haarlem 12/105 (11.4%), Cameroon 5/105 (4.8%), S 3/105 (2.9%), Uganda I 3/105 (2.9%), and X 1/105 (0.95%) (Figures 1, 2). MTBC LAM strains were more diverse in comparison with Ghana strains, while Haarlem strains were not well defined in a branch (Figure 1).

### Comparison of population structure of MTBC strains from Esmeraldas province, Ecuador, and Colombia

The geographic distribution of MTBC strains and lineage distribution for Esmeraldas province, Ecuador, and Colombia are detailed in Figure 2.

The 24-loci MIRU-VNTR patterns of 385 MTBC strains from Ecuador from the years 2012–2016 were retrieved from our previous studies (9, 29) and included in this analysis (Figure 2). The lineage distribution for the MTBC strains from Ecuador (excluding Esmeraldas province) was as follows: 5 of 385 Lineage 2–sublineage Beijing (1.3%) and 380 of 385 Lineage 4 (98.7%). Within Lineage 4: 173 of 385 LAM (44.9%), 117 of 385 Haarlem (30.4%), 27 of 385 Ghana (7%), 26 of 385 S (6.7%), 18 of 385 Cameroon (4.7%), 17of 385 Uganda I (4.4%), 1 of 385 NEW-1 (0.26%), and 1 of 385 (0.26%).

Spoligotyping and 24-loci MIRU-VNTR information of 190 MTBC strains from Colombia from years 2012–2014 was retrieved from reference 4, obtaining the following MTBC lineage distribution: 2 of 190 Lineage 1–Sublineage Manu (1.1%); 20 of 190 Lineage 2–Beijing (10.5%); 158 of 190 Lineage 4 (83.2%). Within Lineage 4: 71 of 190 LAM (37.4%); 44 of 190 Haarlem (25.8%); 18 of 190 T (9.5%); 14 of 190 X (7.4%); 5 of 190 S (2.6%); 1 of 190 Cameroon (0.52%); and 15 of 190 were of unknown lineage (Figure 2).

**Figure 3:**
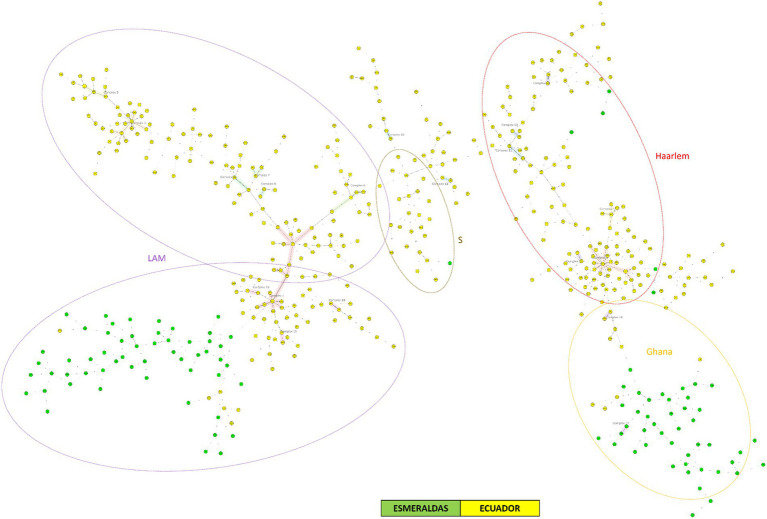
Minimum spanning tree of MTBC strains from Ecuador and Esmeraldas included in this study using 24-loci MIRU-VNTR data. Distinction of genotypic lineages is shown by dotted circles of different colors. The maximum locus difference within a clonal complex is 2.

Figure 3 represents the Minimum Spanning Tree (MST) for MTBC strains from Ecuador (2012–2016) and Esmeraldas (2012–2016) using 24-loci MIRU-VNTR information. This analysis included a total number of 495 MTBC strains, including the 105 MTBC strains for Esmeraldas province (See also de NJT in Supplementary Figure S1). This province is highly represented in this analysis as the number of MTBC strains from the most populated provinces of Pichincha and Guayas were 155 and 59, respectively. A very strong segregation of 99 out of the 105 MTBC strains from Esmeraldas compared to the rest of the provinces of Ecuador was observed. Within those 99 strains, two big and clearly delimitated groups of MTBC strains belonging to sublineage LAM and Ghana were found. Only one clonal complex was observed from MTBC strains from Esmeraldas, including exclusively 2 MTBC strains sublineage Ghana from Esmeraldas, with a maximum of 2 loci difference in the 24 MIRU-VNTR pattern (Figure 3). On the other hand, six MTBC strains from Esmeraldas are clearly dispersed within the MTBC strains from the rest of Ecuador. However, none of the 19 clonal complexes that occur in different groups of lineages of MTBC strains from Ecuador included MTBC strains from Esmeraldas.

**Figure 4:**
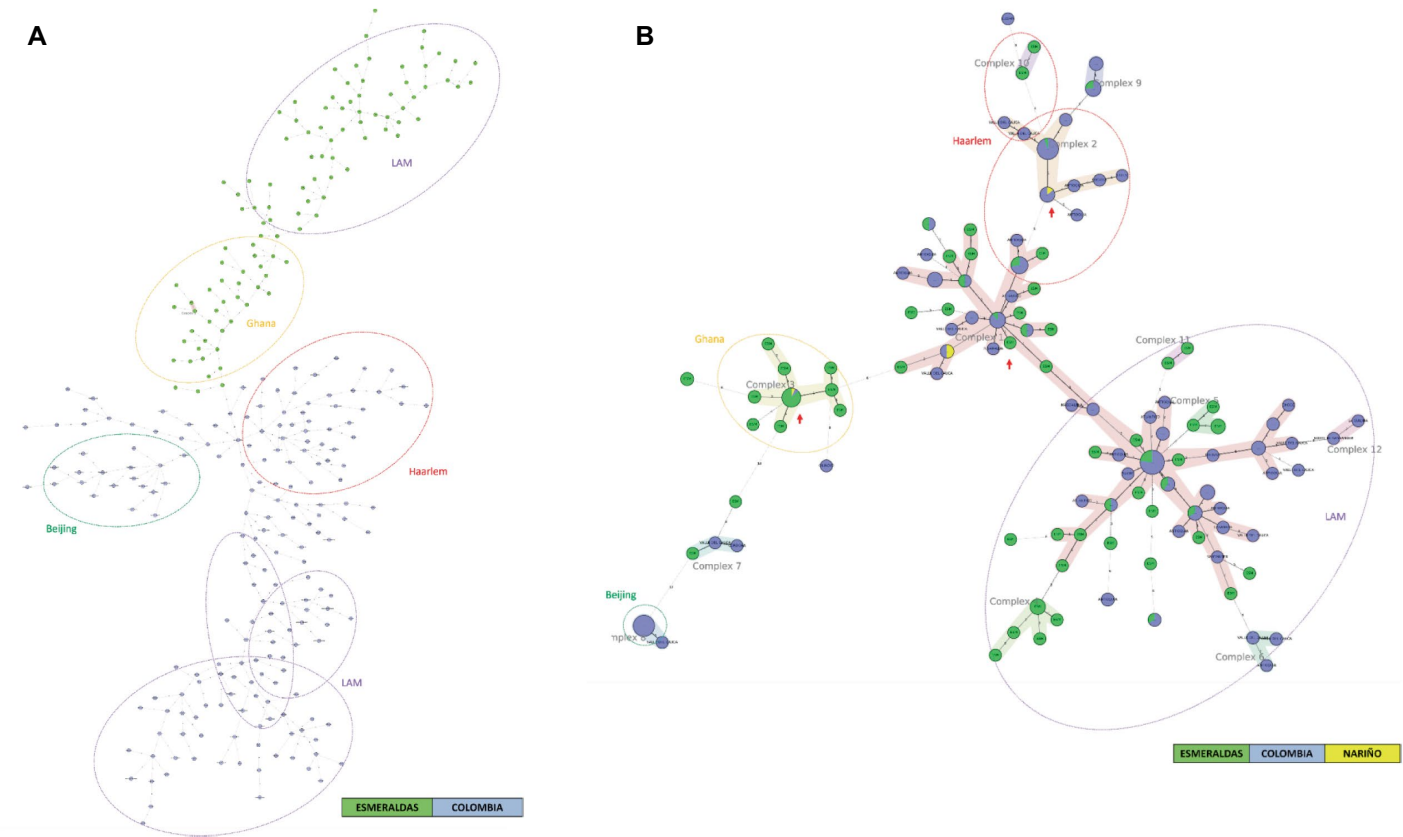
Minimum spanning trees of MTBC strains from Esmeraldas and Colombia included in this study: **(A)** using 24-loci MIRU-VNTR data and **(B)** using Spoligotyping data. Distinction of genotypic lineages is shown by dotted circles of different colors. Red arrows indicate clonal complexes formed between MTBC strains from Esmeraldas province and Colombia (Maximum locus difference within a clonal complex is 2). MTBC strains from the Colombian Department of Nariño, on the border with Esmeraldas, are labeled in yellow.

Figure 4A represents the minimum spanning tree (MST) for MTBC strains from Colombia (2012–2014) and Esmeraldas (2012–2016) using 24-loci MIRU-VNTR information. This analysis included a total number of 190 MTBC strains from Colombia and 105 strains from Esmeraldas province. There is a strong segregation between all the MTBC strains of Esmeraldas and Colombia in the MST, without any clonal complex including strains from both locations (Figure 3). A consistent result was obtained for the NJT (Supplementary Figure S2) where only 2 MTBC strains from Esmeraldas belonging to sublineage Haarlem and LAM were found more phylogenetically related to MTBC strains from Colombia than to other strains from Esmeraldas, both not clustering in clonal complexes.

Figure 4B represents the Minimum Spanning Tree (MST) for MTBC strains from Colombia (2012–2015) and Esmeraldas (2012–2016) using only spoligotyping information. Within the reduced segregation capacity of the spoligotyping method for active transmission events, five well-defined clonal complexes (CC1, CC2, CC3, CC7, and CC9) of L4 sublineages including MTBC strains from Esmeraldas and Colombia were observed. Interestingly, three of those clonal complexes (CC1, CC2, and CC3) included MTBC strains from the department of Nariño in the south of Colombia on the border with Esmeraldas and Carchi provinces in Ecuador; isolates reported from this department share three clonal complexes with strains from Esmeraldas (CC1, CC2, and CC3).

## Discussion

The rapid identification of a highly active transmission complex of MTBC is crucial to reinforce TB surveillance and control programs. Colombia is one of the countries in Latin America that reports a high burden of MTB of lineages commonly associated with antibiotic resistance and increased transmission (4, 7, 13, 15, 23, 24). We addressed the potential transmission of high-risk MTBC strains from Colombia to Ecuador through the identification of the dominant MTBC genotypes in the border province of Esmeraldas.

The population structure of MTBC in the province of Esmeraldas showed a high genetic variability itself, as no MTBC active transmission clusters were found in the collection of 105 strains for 2014–2016. Additionally, most MTBC strains from Esmeraldas were not phylogenetically closely related to strains from the other Ecuadorian provinces, as no mixed clonal complexes were found. A high level of variability within a reduced rural and isolated geographical area has been also described in Panama and Ecuador (29, 37), and the lack of active transmission clusters despite the high burden of TB could be explained as a consequence of relapse of latent tuberculosis cases (9, 29, 37). Interestingly, the MTBC lineage distribution in Esmeraldas is exclusively composed of MTBC lineage 4 strains with no presence of Lineage 2–sublineage Beijing. Despite the close proximity to Colombia where Lineage 2–sublineage Beijing represents 5% of the MTBC population (4, 24), we could not find evidence of recent transnational transmission of this lineage. These results confirm previous findings reporting circulation of Beijing lineage only in limited locations of the Americas like Cuba, Colombia, and Peru (24). Nevertheless, permanent genetic surveillance should be implemented within the Ecuadorian national TB surveillance program to identify active transmission clusters, as has been described, for instance, in Panama (37) or Tunisia (27, 28). This is especially relevant considering the presence of hot spots for active transmission clusters like prisons in Latin American region (38).

Regarding drug resistance results, we found a 16.3% prevalence of MDR-TB in our study population. This value is much higher than the 4.35% MDR-TB prevalence estimated for Ecuador by the WHO (1, 8). However, similar results of higher prevalence of MDR-TB that WHO estimation was also found in the previous study for the whole country (9). This difference could be explained by a bias in the TB samples that are received by the National Reference Laboratory in Ecuador. In this sense, not all TB patient samples are processed but those defined by a triage protocol that includes all the samples with drug treatment failure, increasing the probability of detecting MDR-TB.

This study has some limitations. First, the 105 MTBC strains included in the study represented less than 20% percent of total TB cases in Esmeraldas (see details in methods), so we cannot totally rule out the presence of Beijing lineage or active transmission cluster due to sampling bias. Second, as we could only access MTBC strains from 2014 to 2016, the current TB transmission scenario could have changed, especially considering the high level of migration back and for across the Ecuador–Colombia border in recent years due to the huge humanitarian and migratory crisis following the economic collapse of Venezuela (39). Third, the phylogenetic analysis had different resolutions depending on the data available: While all the MTBC strains from Esmeraldas and Colombia had 24-loci MIRU-VNTR and spoligotyping data available, the MTBC strains from other provinces of Ecuador lacked spoligotyping information. Fourth, either MTBC strains collections from Colombia and Ecuador could also have geographical bias, as most of those strains came from the Department of Valle del Cauca in Colombia and from the main cities of Ecuador (Quito and Guayaquil) (4, 9). Moreover, the lack of MTBC strains from the other Ecuadorian border provinces with Colombia represents another source of potential sample bias.

In conclusion, cheap and easy molecular epidemiology tools are still useful for middle to high-burden TB settings in Latin America where whole-genome sequencing is still an expensive approach. Further studies with greater MTBC sample sizes from recent years in this region and others in Ecuador are needed to confirm the main findings in our study, so they could be considered for improvements in the TB surveillance program in high-burden rural settings like Esmeraldas.

## Data availability statement

The original contributions presented in the study are included in the article/Supplementary materials, further inquiries can be directed to the corresponding author.

## Ethics statement

This study was authorized by the Institutional Review Board of Universidad de Las Américas (code 2024-EXC-001). Written informed consent for participation was not required from the participants or the participants’ legal guardians/next of kin in accordance with the national legislation and institutional requirements.

## Author contributions

BC-R: Conceptualization, Data curation, Formal analysis, Investigation, Methodology, Validation, Writing – original draft, Writing – review & editing. SE-A: Data curation, Formal analysis, Investigation, Methodology, Writing – review & editing. GF-S: Conceptualization, Funding acquisition, Investigation, Project administration, Resources, Validation, Writing – review & editing. JB-M: Data curation, Methodology, Validation, Writing – review & editing. NJ-P: Formal analysis, Investigation, Methodology, Validation, Writing – review & editing. CC-F: Data curation, Formal analysis, Investigation, Methodology, Validation, Writing – review & editing. JG: Data curation, Formal analysis, Investigation, Methodology, Writing – review & editing. JJ: Funding acquisition, Resources, Validation, Writing – review & editing. JH: Conceptualization, Funding acquisition, Investigation, Project administration, Resources, Writing – review & editing. MG-B: Conceptualization, Data curation, Formal analysis, Funding acquisition, Investigation, Project administration, Resources, Supervision, Validation, Writing – original draft, Writing – review & editing. SO: Conceptualization, Resources, Writing – review & editing.

## Footnotes

^1^https://www.salud.gob.ec/gacetas-tuberculosis/

^2^https://www.miru-vntrplus.org
