# Supplementary material for: A first insight into tuberculosis transmission at the border of Ecuador and Colombia: a retrospective study of the population structure of Mycobacterium tuberculosis in Esmeraldas province
Source: Front Public Health. 2024 Feb 7;12:1343350. doi: 10.3389/fpubh.2024.1343350 (PMC10879341; doi:10.3389/fpubh.2024.1343350)
Supplement: Supplementary file 1 [file Table_1.docx]

**Supplementary Table 1.** Distribution by country and province/department of MTBC strains from Ecuador and Colombia included in this study.

| **ECUADOR^1^** | | **COLOMBIA^2^** | | |
| --- | --- | --- | --- | --- |
| **Province** | **Samples** | **Department** | **Samples** | **Source** |
| Azuay | 8 | Antioquia | 67 | Rodríguez-Castillo *et al.*, 2020 |
| Bolívar | 2 | Atlántico | 9 |  |
| Cañar | 5 | Bogotá | 4 |  |
| Chimborazo | 2 | Bolívar | 5 |  |
| Cotopaxi | 1 | Boyacá | 1 |  |
| **Esmeraldas** | **105** | Caldas | 1 |  |
| Galápagos | 1 | Casanare | 2 |  |
| Guayas | 155 | Cauca | 1 |  |
| Los Ríos | 44 | Cesar | 3 |  |
| Manabí | 12 | Chocó | 6 |  |
| Morona Santiago | 1 | Córdoba | 2 |  |
| Napo | 3 | Huila | 2 |  |
| Orellana | 1 | La Guajira | 3 |  |
| El Oro | 56 | Magdalena | 1 |  |
| Pastaza | 2 | Meta | 1 |  |
| Pichincha | 59 | Nariño | 4 |  |
| Santo Domingo de los Tsáchilas | 8 | Norte de Santander | 7 |  |
| Santa Elena | 11 | Putumayo | 4 |  |
| Sucumbíos | 10 | Quindío | 4 |  |
| Tungurahua | 2 | Risaralda | 7 |  |
| Zamora Chinchipe | 2 | Santander | 3 |  |
| **TOTAL** | **490** | Sucre | 2 |  |
|  |  | Tolima | 3 |  |
|  |  | Valle del Cauca | 48 |  |
|  |  | **TOTAL** | **190** |  |
| ^1^ Ecuadorian samples were collected between the years 2012-2016  ^2^ Colombian samples were collected between the years 2012-2013 | | | | |
