# Supplementary material for: A first insight into tuberculosis transmission at the border of Ecuador and Colombia: a retrospective study of the population structure of Mycobacterium tuberculosis in Esmeraldas province
Source: Front Public Health. 2024 Feb 7;12:1343350. doi: 10.3389/fpubh.2024.1343350 (PMC10879341; doi:10.3389/fpubh.2024.1343350)
Supplement: Supplementary file 2 [file Data_Sheet_1.docx]

| 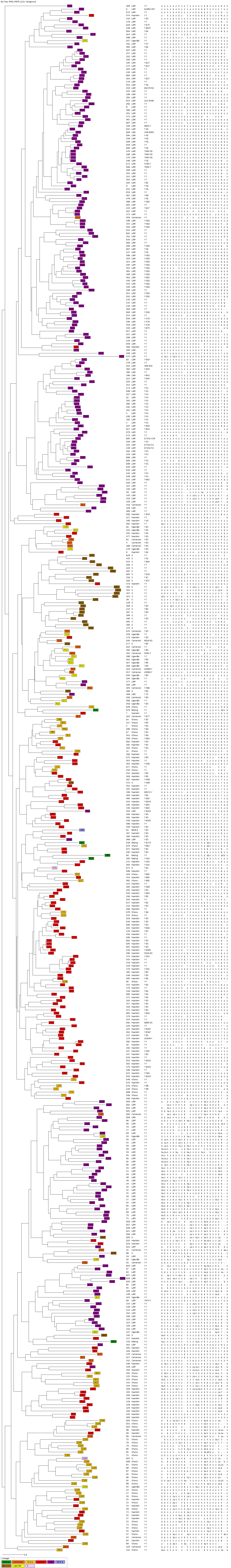 | | **Supplementary Figure 1.** Neighbour-joining Tree of MTBC strains from Ecuador included in this study, using 24-loci MIRU-VNTR information. |
| --- | --- | --- |
| 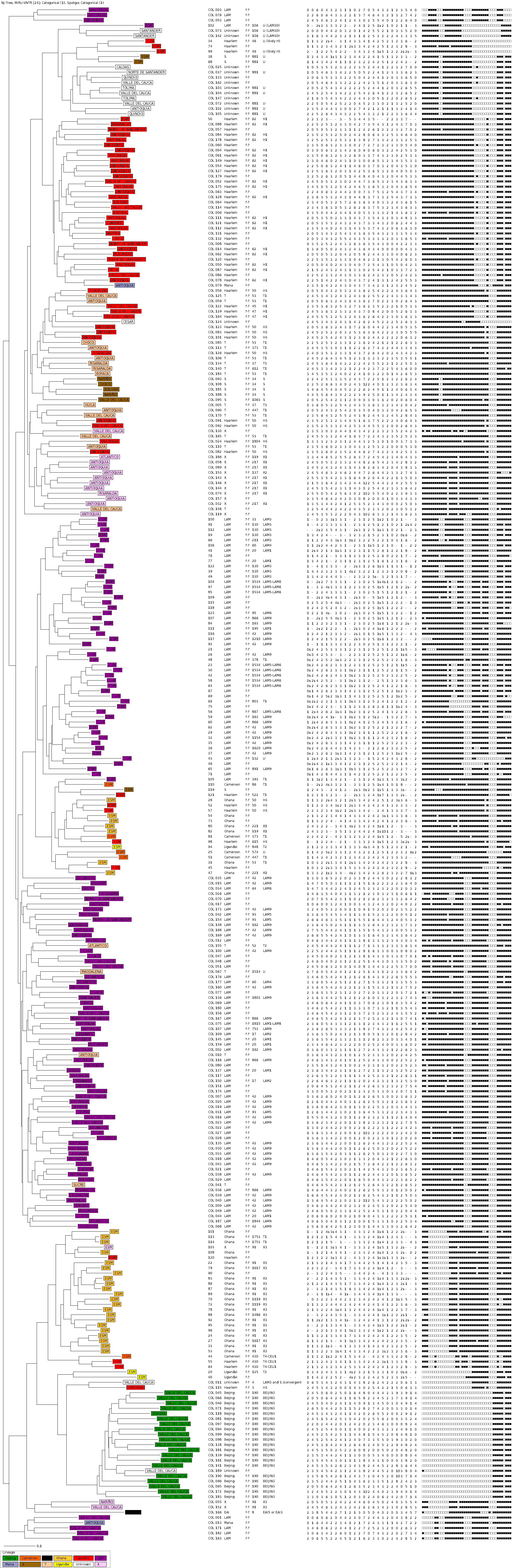 | **Supplementary Figure 2.** Neighbour-joining Tree of MTBC strains from Esmeraldas province and Colombia included in the study. Spoligotyping and 24-loci MIRU-VNTR information was used for the construction of the dendrogram. | |
